# Global pattern of plant utilization across different organisms: Does plant apparency or plant phylogeny matter?

**DOI:** 10.1002/ece3.2882

**Published:** 2017-03-14

**Authors:** Xiaohua Dai, Wei Zhang, Jiasheng Xu, Kevin J. Duffy, Qingyun Guo

**Affiliations:** ^1^ Leafminer GroupSchool of Life and Environmental SciencesGannan Normal UniversityGanzhouChina; ^2^ National Navel Orange Engineering Research CentreGanzhouChina; ^3^ Institute of Systems ScienceDurban University of TechnologyDurbanSouth Africa; ^4^ School of MathematicsStatistics and Computer ScienceUniversity of KwaZulu‐NatalDurbanSouth Africa

**Keywords:** consumer resource, ethnobotany, host islands, plant apparency, plant phylogeny, plant utilization

## Abstract

The present study is the first to consider human and nonhuman consumers together to reveal several general patterns of plant utilization. We provide evidence that at a global scale, plant apparency and phylogenetic isolation can be important predictors of plant utilization and consumer diversity. Using the number of species or genera or the distribution area of each plant family as the island “area” and the minimum phylogenetic distance to common plant families as the island “distance”, we fitted presence–area relationships and presence–distance relationships with a binomial GLM (generalized linear model) with a logit link. The presence–absence of consumers among each plant family strongly depended on plant apparency (family size and distribution area); the diversity of consumers increased with plant apparency but decreased with phylogenetic isolation. When consumers extended their host breadth, unapparent plants became more likely to be used. Common uses occurred more often on common plants and their relatives, showing higher host phylogenetic clustering than uncommon uses. On the contrary, highly specialized uses might be related to the rarity of plant chemicals and were therefore very species‐specific. In summary, our results provide a global illustration of plant–consumer combinations and reveal several general patterns of plant utilization across humans, insects and microbes. First, plant apparency and plant phylogenetic isolation generally govern plant utilization value, with uncommon and isolated plants suffering fewer parasites. Second, extension of the breadth of utilized hosts helps explain the presence of consumers on unapparent plants. Finally, the phylogenetic clustering structure of host plants is different between common uses and uncommon uses. The strength of such consistent plant utilization patterns across a diverse set of usage types suggests that the persistence and accumulation of consumer diversity and use value for plant species are determined by similar ecological and evolutionary processes.

## 1 Introduction

There are over 300,000 plant species globally (including flowering plants, gymnosperms, ferns and allies, bryophytes and plant algae) (Mora, Tittensor, Adl, Simpson, & Worm, 2011). Plants make up more than 99% of the total living matter in the world and are the ultimate energy source for most of the life on Earth (Keddy, 2007). However, different plants are not equally utilized by pathogens, parasites, insects, animals, or humans (Miller, 2012). Which plants are more likely to be used by humans or animals and which plants support more herbivores and pathogens are fundamental questions in ecology and ethnobiology.

### 1.1 Definition of plant apparency

Plant apparency, or plant commonness, is an important indicator of the utilization of plant resources (Feeny, 1976; Guèze et al., 2014). The plant apparency hypothesis implies that more apparent plants suffer more herbivory and, thus, invest more in quantitative chemical defenses (Feeny, 1976; Smilanich, Fincher, & Dyer, 2016; Soldati, de Medeiros, Duque‐Brasil, Coelho, & Albuquerque, 2016; Strauss, Cacho, Schwartz, Schwartz, & Burns, 2015). Such chemical weapons may reduce the number of specific enemies but not completely eliminate enemy attack. Moreover, these defensive compounds can have positive effects on herbivore performance (Smilanich et al., 2016). Plant commonness could facilitate the evolutionary adaptation of enemies, and many enemies will use such defensive compounds to locate host plants (Smilanich et al., 2016).

Other authors explain apparency from the perspective of observed host use by animals (Chew & Courtney, 1991). The concept of apparency has also been extended to ethnobiology, where humans are treated as foragers or consumers, similar to nonhuman herbivores (Lozano, Araújo, Medeiros, & Albuquerque, 2014; de Lucena, de Lima Araújo, & de Albuquerque, 2007). As a variant of the plant apparency hypothesis, the ecological apparency hypothesis implies that humans tend to collect and use apparent plants, similar to insects and other organisms (Phillips & Gentry, 1993; Ribeiro et al., 2014; Soldati et al., 2016). Apparent plants are more likely to be found by parasites, natural enemies, pollinators and humans (Feeny, 1976; Phillips & Gentry, 1993; Schlinkert et al., 2015), while rare plants are difficult to find or become profitless and therefore escape enemies (i.e., the rare species advantage hypothesis) (Bachelot & Kobe, 2013; Chew & Courtney, 1991; Parker et al., 2015). According to the optimal foraging strategy, more available species should be preferred because they are easier to discover and should therefore reduce time and energy costs (Gonçalves, Albuquerque, & de Medeiros, 2016).

Hence, apparency indicators can be divided into two major categories (Table 1): (1) quantitative availability, which increases the random encounter rate between consumers and plants, related to either random searching or active searching by parasites (e.g., the abundance, spatiotemporal distribution, or biomass of a given plant species); and (2) qualitative detectability, which makes plants visually or chemically distinct from their background and is related to consumers’ perceptual abilities and feeding habits (Castagneyrol, Giffard, Péré, & Jactel, 2013; Courtney, 1982; Schlinkert et al., 2015; Strauss et al., 2015; Wiklund, 1984) (e.g., the odor, color, plant composition or background environment of a given plant species). The ecological apparency hypothesis in ethnobiology (Phillips & Gentry, 1993) focuses more on quantitative apparency, while the plant apparency hypothesis in insect ecology (Feeny, 1976) focuses more on qualitative apparency and plant chemical defenses. Quantitative apparency will generally increase attacks from enemies, while the roles of qualitative apparency are complicated. For example, specific plant defensive compounds may reduce visiting and feeding by most insects but attract some herbivore specialists (Smilanich et al., 2016), and red leaf color is a warning signal for many animals, but there are some exceptions (Stutz *et al*., 2016). However, the total apparency of one plant is the combination of all quantitative and qualitative apparency indicators and functions as a whole in relation to enemies.

**Table 1:** Indicators of plant apparency for susceptibility to encounter by parasites

Indicator of plant apparency	Degree of plant apparency	Selected references
Abundance	High abundance > low abundance	Feeny (1976); Hay (2016)
Density	High density > low density	de Albuquerque and de Lucena (2005); de Lucena, de Medeiros, Araújo, de L Alves, and de Albuquerque (2012); de Lucena et al. (2007); Strauss and Cacho (2013)
Frequency	High frequency > low frequency	de Albuquerque and de Lucena (2005); de Lucena et al. (2007, 2012)
Spatial distribution	Wide distribution > narrow distribution; clumped distribution > random distribution; larger patch size > small patch size	Compton and Hawkins (1992); Joy and Crespi (2012); Kareiva (1985); Leather (1986)
Temporal distribution	Long duration > short duration; predictable > unpredictable	Castagneyrol et al. (2013); Feeny (1976); Hay (2016); Lawton (1983); Stanton et al. (2016); Strauss and Cacho (2013); Strauss et al. (2015)
Body size	Large plant > small plant	Feeny (1976); Hay (2016); Lawton (1983); Strauss and Cacho (2013)
Height	Tall plant > short plant	Castagneyrol et al. (2013); Lawton (1983); Strauss et al. (2015)
Dominance	High dominance > low dominance	(de Albuquerque and de Lucena (2005); Hay (2016); de Lucena et al. (2007, 2012)
Importance value	High importance value > low important value	de Albuquerque and de Lucena (2005); de Lucena et al. (2007, 2012)
Life form	Tree > herb	Feeny (1976); Lawton (1983); Stanton et al. (2016); Strauss and Cacho (2013); Strauss et al. (2015)
Chemical signals (e.g., odor, taste)	Attracting plant > deterring plant; palatable plant > unpalatable plant	Chew and Courtney, (1991); Ernest, (1989); Euler and Baldwin (1996); Parmesan (1991); Stutz *et al*. (2016); Stanton et al. (2016); Strauss and Cacho (2013)
Visual signals	Visible plant > invisible plant	Niu et al. (2014); Stutz *et al*. (2016); Strauss and Cacho (2013)
Alternative diversity within the target plant group (e.g., genotypes, phenotypes, ecotypes)	High diversity > low diversity	McArt and Thaler (2013); Utsumi, Ando, Craig, and Ohgushi (2011)
Neighbors of target plants	Differences between a focal plant and neighbors: Same taxa > different taxa; close relatives > distant relatives	Castagneyrol et al. (2013); Moreira, Abdala‐Roberts, Parra‐Tabla, and Mooney, (2014); Stanton et al. (2016)
Background environment	Sparsely vegetated environment > densely vegetated environment; simple environment > complex environment	Lopresti and Karban (2016); Strauss and Cacho (2013); Strauss et al. (2015)

In the present study, we focused on only quantitative measurements of plant spatiotemporal availability that are objective and “ultimate,” without reference to the detective abilities of relevant organisms for particular hosts (Courtney, 1985; Rhoades, 1979) or the degree of differences in the searching environments (Strauss et al., 2015).

### 1.2 Parasite patterns on different host plant islands

If we consider host plants to be analogous to islands (Janzen, 1968; Joy & Crespi, 2012; Miller, 2012), species–area relationships or species–distance relationships can be adopted to describe the incidence or richness of parasites on hosts. The “area” in species–area relationships can refer to any quantitative apparency indicator, such as the number of individuals, distribution range, body size, species number, or total biomass of a host taxon (Feeny, 1976; Joy & Crespi, 2012; Kamiya, O'Dwyer, Nakagawa, & Poulin, 2014; Miller, 2012). Higher apparency is associated with more host–parasite encounters (random placement hypothesis) and more niches for parasites (habitat diversity hypothesis) (Miller, 2012; Strona & Fattorini, 2014).

The distances employed in host island studies include geographical distance, phylogenetic distance, environmental distance, and other distance measurements (Joy & Crespi, 2012; Locke, Mclaughlin, & Marcogliese, 2013; Nakadai & Murakami, 2015). Close, but uncommon relatives of common plants are occasionally utilized due to their similarities in terms of chemical constituents. Therefore, we should also consider the effects of plant phylogeny on plant‐use patterns (Ødegaard, Diserud, & Østbye, 2005; Parker et al., 2015). In general, phylogenetically close hosts tend to harbor similar parasites or pathogens because of similarities in their evolutionary histories and ecological characteristics (Grandez‐Rios, Bergamini, De Araujo, Villalobos, & Almeida‐Neto, 2015; Joy & Crespi, 2012; Nakadai & Murakami, 2015; Pearse & Hipp, 2009). A decay of parasite similarity, richness, specialization and performance with host phylogenetic distance (distance decay hypothesis) has been observed in some host–parasite systems (Branco, Brockerhoff, Castagneyrol, Orazio, & Jactel, 2015; Grandez‐Rios et al., 2015; Locke et al., 2013; Nakadai & Murakami, 2015; Novotny et al., 2012).

Studies on whether a certain plant is used by humans or other organisms are generally performed at local or regional scales (Brändle & Brandl, 2001; Guèze et al., 2014). In contrast, global‐level studies are scarce but are important for evaluating whether plant apparency or plant phylogeny can predict patterns of plant use across different organisms. Moreover, the present study may be the first to consider human and nonhuman consumers together in the analysis of plant utilization patterns. We will prove that at a global scale, plant apparency and phylogenetic isolation can be important predictors of plant utilization and consumer diversity.

## 2 Materials and Methods

### 2.1 Data collection

The main sources of global plant utilization data used in the present study included review articles, monographs, professional databases and specialized websites addressing plant uses (Appendix S1). As some sources may be outdated or incomplete, we also employed the ISI Web of Science^™^ (WoS) to obtain more plant utilization data based on keyword searches (Appendix S1). For example, to study the host plants of Agromyzid flies, we obtained an initial host plant list from the book “Host Specialization in the World Agromyzidae (Diptera)” published in 1990 and then searched WoS publications from 1990 to 2015 using the following search terms: Topic: (Agromyzidae) AND Topic: (host plant*). However, when too many hits were obtained in WoS (>500 hits), we narrowed the search terms by replacing TS (Topic) with TL (Title) and so on (this seldom occurred). We then manually checked and extracted host plant names article by article. The deadline for all utilization data was 31 December 2015. The literature search using WoS was similar to increase the sampling effort in field investigations; however, few additional host families were identified through the WoS search and most of those families were small (Appendix S1). Thus, even without the WoS search, the general patterns observed in the present study were consistent and were confirmed by our previous data analyses.

Plant names (species, genera, families, or mixtures of the three levels) were checked and resolved using the Taxonomic Name Resolution Service, v 4.0 (Boyle et al., 2013) and were verified with The Plant List, v 1.1 (http://www.theplantlist.org/). Then, we summarized the list of matched and accepted family names for each utilization group. We focused on angiosperm plants in this study only because many utilizers, such as pollinators and Tischeriidae, seldom use ferns and gymnosperm. The names of 420 angiosperm families (Parker et al., 2015) were obtained according to the APG III system (The Angiosperm Phylogeny Group, 2009), which updates the Angiosperm Phylogeny Website (http://www.mobot.org/MOBOT/research/APweb). We added some new host plant family data for Tischeriidae and the leaf‐mining Chrysomelidae based on our fieldwork and laboratory research. We also included datasets for different human uses and datasets for plant sexual systems as indirect indicators of different pollinator combinations. Some utilization groups (e.g., Cercopoidea among sap suckers) were not employed because the number of associated host plant families was less than 10 (<2.5% of the total number of plant families), which may bias the following analyses of either phylogenetic signaling or binomial GLM (generalized linear model) fitting.

### 2.2 Plant families as islands

To compare the plant utilization patterns among human and nonhuman consumers, we employed presence–absence data as consumer characteristics and plant families as islands. Presence–absence data for plant utilization at the plant family level is easy to obtain, while the collection costs for abundance data for plant utilizers at a plant species level are high, making it impossible to obtain such data, especially at larger spatial scales. Presence–absence data are more appropriate for clarifying the effects of host characteristics on parasite similarity (Locke et al., 2013), especially at broad scales (i.e., continental to global scales).

Compared with the species level, ecological associations at the family level may exhibit more fundamental and reliable characteristics (Hamm & Fordyce, 2015; Ødegaard et al., 2005; Ricklefs, 1987; Ward, Hackshaw, & Clarke, 1995; Ward & Spalding, 1993). Possible reasons for this difference include the following: (1) the origins of families are more ancient (Ricklefs, 1987); (2) the detailed complications of variability among families are reduced (Ward et al., 1995); and (3) the bias of collection and identification at the family level is lower (Hamm & Fordyce, 2015; Ward & Spalding, 1993). There are many plant species names that remain unresolved in The Plant List, v 1.1, and it is quite difficult to obtain a full list of utilized plants at the species level for the present day due to a lack of sampling effort around the world. However, focusing on the utilized plants at the family level provides a more easily obtainable and nearly complete list because the sampling pool of angiosperm plant families in relation to angiosperm species is approximately 420:200,000 = 0.21%.

Previous studies have shown significant effects of plant taxonomy at the family level on parasite abundance (Menken, Boomsma, & van Nieukerken, 2010; Olsson‐Pons, Clark, Ishtiaq, & Clegg, 2015; Szendrei & Rodriguez‐Saona, 2010). The host ranges of nearly all herbivore groups, except for the most polyphagous, are limited to plants at the genus or family level (Doorenweerd, Van Nieukerken, & Menken, 2015; Ødegaard et al., 2005; Pearse, Harris, Karban, & Sih, 2013; Weiblen, Webb, Novotny, Basset, & Miller, 2006). Herbivore similarity decreases obviously from the host species/genus level to the host family level (Ødegaard et al., 2005; Weiblen et al., 2006). The identity of defensive allelochemicals may be phylogenetically conserved at the plant family level (Barton & Koricheva, 2010; Ehrlich & Raven, 1964; Strauss et al., 2015; Szendrei & Rodriguez‐Saona, 2010). Some parasites can discriminate host plants according to plant traits at the family level (Ricklefs, 2008).

If we regard a plant species as the island, the plant's genus or family is like an archipelago of species islands (Janzen, 1968). The above species–area, species–apparency and species‐distance–relationships should also be true at the archipelago level. Such relationships between parasites and host plant families have been discovered in galling insects (Joy & Crespi, 2012; Price, 1977) and other insects (Lill, Marquis, & Ricklefs, 2002). The plant family size hypothesis indicates that larger plant families are expected to be associated with more insect species than smaller families (Araújo, 2011; de Araújo, dos Santos, & Gomes‐Klein, 2012; de Araújo, Silva, dos Santos, & Gomes‐klein, 2013; Cuevas‐Reyes, Quesada, Hanson, & Oyama, 2007; Fernandes, 1992; Gonçalves‐alvim, Fernandes, & Goncalves‐Alvim, 2001; Lawton & Price, 1979; Mendonça, 2007; Price, 1977; Veldtman & McGeoch, 2003; Ward & Spalding, 1993). The existence of more species in a given family corresponds to more available niches for parasites (de Araújo et al., 2012, 2013; Joy & Crespi, 2012; Milton de Souza Mendonça, 2007). However, the relationship between parasite richness and plant genus size is weaker than that for plant family size (Araújo, 2011; de Araújo et al., 2012).

### 2.3 Plant phylogeny

We constructed a phylogenetic supertree (Appendix S2) with ages for all vascular plant families based on the R2G2_20140601 super tree (Parker et al., 2015) using Dendroscope 3.2.10 (Huson & Scornavacca, 2012). We deleted within‐family topological structures from the R2G2_20140601 tree because our study focused on only the family level. We tested the degree of the phylogenetic signal (Fritz & Purvis, 2010) to measure the presence–absence of utilization of a single plant family in each utilization group. The *D* statistic is an estimate of phylogenetic structure with binary values: when *D *=* *0, there is phylogenetic clumping under Brownian motion; *D *=* *1 corresponds to no (random) phylogenetic signal; *D* > 1 indicates phylogenetic overdispersion; and *D* < 0 represents high phylogenetic conservation (Fritz & Purvis, 2010). We used the phylo.d function in the R package caper (https://cran.r-project.org/package=caper) to estimate the *D* statistics and their associated *p*‐values (*p*_random_ and *p*_Brownian_) with 1,000 permutations. We employed linear correlation to analyze the relationship between *D* and the number of host plant families for each utilization group (Appendix S3).

### 2.4 Plant apparency

In this study, we regarded the numbers of species and genera and the distribution area of a single plant family as plant apparency. The species number and genus number can represent niche diversity in a plant family, while the distribution area determines the encounter rate between each consumer and the target plant family. We obtained the numbers of species and genera in each plant family from The Plant List, and only accepted names were accounted for. Families listed in The Plant List without an accepted name were assigned a species number and genus number of 0.5. Families that were not listed in The Plant List were assigned a species number and genus number according to the Angiosperm Phylogeny Website. Sketch maps for the distribution of each plant family were obtained from the Angiosperm Phylogeny Website, and the rough terrestrial distribution area (km^2^) of each plant family was calculated using ImageJ 1.48v (Schneider, Rasband, & Eliceiri, 2012) based on a pixel number‐area transformation (Appendix S4). Note that the distribution maps for some plant families were merged from sketch maps of within‐family groups. For some small families without a distribution map, we assigned the distribution area a small value of 1,000 km^2^.

### 2.5 Data analyses

The presence–absence of utilized plant families for each utilization group was recorded as either binary data or 0–1 data. Defining the utilization probability (UP) as the probability that the plant family was utilized by one utilization group, 1‐UP was the probability that the plant family was not utilized. The logit of UP was defined as logit(UP) = ln(UP/(1‐UP)). Therefore, we could adopt a binomial GLM with logit link (UP = exp(*a *× PA* *+ *b*)/(exp(*a *× PA* *+ *b*) + 1)) to predict UP as a function of plant apparency (PA; the species number in a plant family, here), where logit(UP) = *a *× PA* *+ *b*;* a* is the slope for measuring the increase in the logit for a one unit increase in PA; and *b* is the intercept. As PA* *→ ∞, UP ↓ 0 when *a *<* *0, and UP ↑ 1 when *a *>* *0 (Agresti, 2002). The *G* statistic (*G *= *a* ^2^/VAR(*a*), *df* = 1) calculated in PAST can measure whether the slope, *a*, is different from 0 (Hammer, Harper, & Ryan, 2001). With the logit link, the GLM does not predict UP values outside of the 0–1 range (Zuur, Ieno, Walker, Saveliev, & Smith, 2009). Moreover, GLM plots are very informative for continuous variables such as PA (Kindt & Coe, 2005). Thus, such binomial logistic regression models are often used to study many binary ecological or evolutionary patterns, such as species occurrence, parasitism risk and disease prevalence (Agresti, 2002; Kindt & Coe, 2005; Zuur et al., 2009).

**Figure 1:**
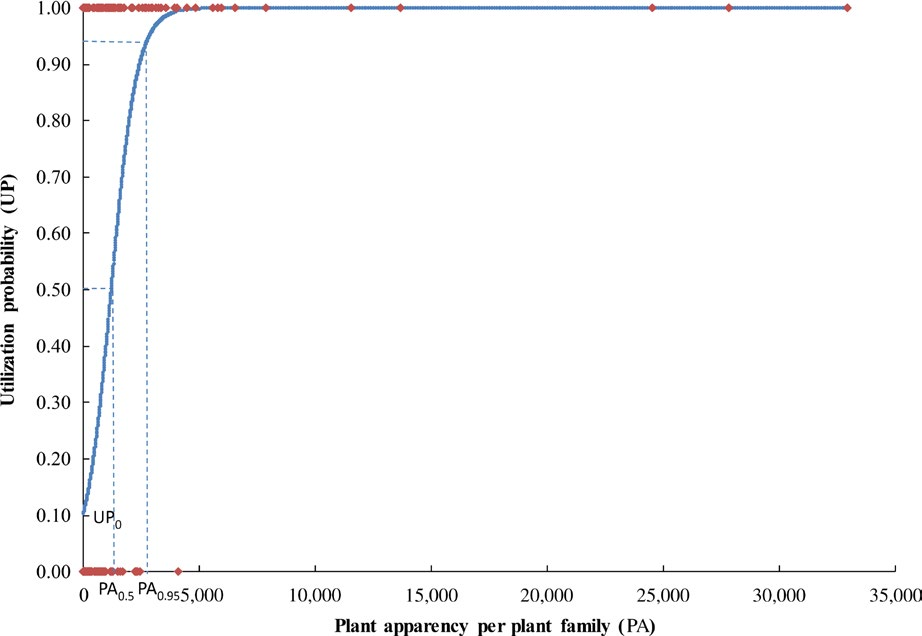
Utilization probability depends on plant apparency. A binomial GLM with a logit link (UP = exp(*a *× PA **+ *b*)/(exp(*a *× PA **+ *b*)* *+ 1), *a *=* *0.0018, *b *=* *−2.15, *p*(*a *=* *0) < .00001, *n* = 420) for predicting the utilization probability (UP) of each plant family as a function of plant apparency (PA; the species number in a plant family, here) for a utilization group (leaf‐mining Chrysomelidae, here). PA_0.5_ is the apparency at which the probability of being utilized is 0.5; PA_0.95_ is the apparency at which the probability of being utilized is 0.95; and UP_0_ is the UP for the most unapparent plant families (PA **→ 0)

We generated 1,000 binomial random samples for 420 plant families with the probability of success in each trial equal to 0.1, 0.3, 0.5, 0.7 or 0.9. We then fitted the generated binary data to plant apparency using the binomial GLM with the logit link. We estimated plant apparency when UP = 0.5 (PA_0.5_) or UP = 0.95 (PA_0.95_) and the UP when PA = 0 (UP_0_) for both the real and generated data (Figure 1; Appendix S3). Here, PA_0.5_ is the apparency at which the probability of being utilized is 0.5; PA_0.95_ is the apparency at which the probability of being utilized is 0.95; and UP_0_ is the UP for the most unapparent plant families (PA* *→* *0). The differences between real and generated data were considered using scatter plots where *X *= number of host plant families (HF), and *Y *= PA_0.5_, PA_0.95_, or UP_0_. For the actual datasets, we performed linear correlation analysis to analyze the relationships between log_10_(PA_0.5_), log_10_(PA_0.95_), or UP_0_ and HF for each utilization group (Appendix S3). The log transformation of PA_0.5_ or PA_0.95_ not only omits unreasonable negative or zero values but also provides clearer associations between variables. Additionally, we fitted linear relationships between UP_0_ and phylogenetic signals (*D*). The utilizer ratio (UR) of one plant family was the ratio of the number of groups that utilized a plant family (= the number of 1s) to the total number of utilization groups (= the total number of both 0s and 1s = 44). A higher UR indicated that more consumers would like to use the plant. The relationships between UR and plant apparency were fitted. The number of utilization groups analyzed here was 44. We calculated pairwise phylogenetic distances (PD in Myr) between all 420 families with the cophenetic function in the R package Picante (Kembel et al., 2010). We then defined the plant families with 10th quantile apparency (i.e., the top 43 families) as common plant families. Such common plant families are analogous to the “mainland” in island biogeography theory. Hence, we could study the distance‐decaying hypothesis between the mainland and islands. The minimum PD to the top 43 plant families (PD_min_) was used to measure the phylogenetic closeness of a given plant family island to the mainland families. The relationships between UR and the PD_min_ were fitted.

Similar patterns of plant utilization could also be derived through the above analyses by replacing plant apparency with the genus number or land distribution area of a plant family. All analyses were performed in R version 3.1.3 (R Core Team, 2016), R studio 0.98.1087 (R Studio Team, 2016), PAST 3.08 (Hammer et al., 2001), and Microsoft Excel 2016 with XL Toolbox (https://www.xltoolbox.net/).

## 3 Results

We listed world plant families utilized by insects, mites, microbes, pollinators, and humans (i.e., different utilizers) based on a meta‐analysis. As predicted, the utilization probability for every utilization group increased significantly with plant apparency (*a > *0, *p*(*a *=* *0) < .05; Figure 1, Appendices S5 and S6) and decreased significantly with plant phylogenetic distance to common plants (*a < *0, *p*(*a *=* *0) < .05; Appendix S7). It appears that common plants are always selected for common uses. As the plants that are primarily used as food for either insects or humans, these plants would be expected to exhibit high abundance and high accessibility (Thomas, Vandebroek, & Van Damme, 2009), and the relatives of the primary plants would also show a high probability of being targeted.

**Figure 2:**
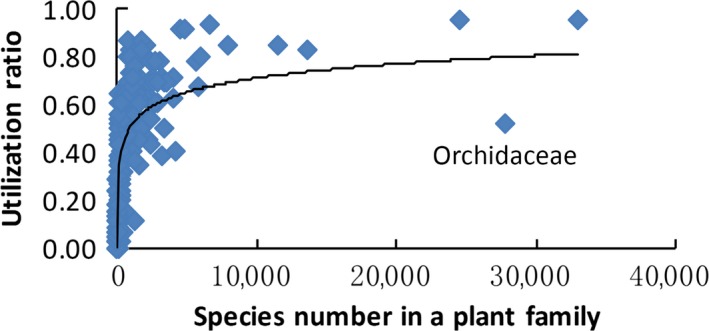
Consumer diversity depends on plant apparency. The utilizer ratio (UR) for each plant family increased with plant apparency (PA; species number in a plant family here) for each utilization group (UR = −0.070 + 0.20 × log_10_(PA), *n *=* *420, *r* ^2^ = .71, *p *<* *.00001)

**Figure 3:**
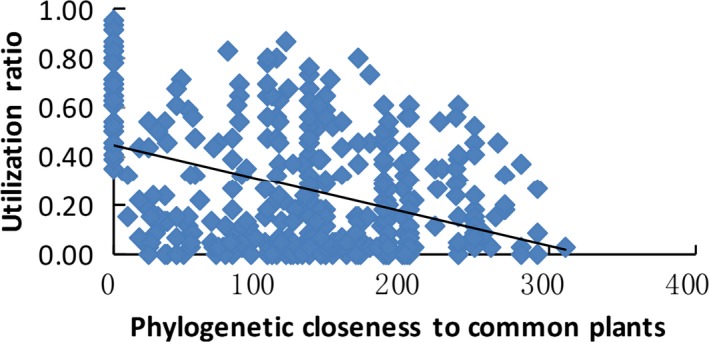
Consumer diversity depends on plant phylogeny. The utilizer ratio (UR) decreased with the phylogenetic distance (PD_min_ in Myr) of each plant family to the nearest common plant family (UR = 0.44 – 0.0014 × PD_min_,* n *=* *420, *r* ^2^ = .16, *p *<* *.00001)

The binomial GLM models were useful for describing the presence–absence of each species on plants with different apparency ratings. This approach is similar to the species–area curves employed in island biogeography, if one regards the plants as islands, apparency as island size and phylogenetic distance as island distance. Generally, apparent and abundant plants supported more utilization groups than unapparent plants (Figure 2), while plants that were phylogenetically close to common plants presented more consumer diversity than phylogenetically distant plants (Figure 3). We refer to the latter phenomenon as an “isolated species advantage” rather than a “rare species advantage.” That is, more consumer diversity is found on larger plant islands or on islands closer to the largest islands, which is consistent with many previous studies involving hosts as islands (Miller, 2012; Parker et al., 2015).

An unexpected exception was found for Orchidaceae (Figure 2), which hosted fewer herbivores and sexual systems than other common plant families. The acceleration of orchid species diversification in history is correlated with, for example, the evolution of pollinia, epiphytic habits and sophisticated insect pollination mechanisms (Givnish et al., 2015). Pollinating predatory wasps, nectary‐attracting bodyguard ants and flowers showing wasp mimicry may play important roles in the protection of orchids from herbivory (Subedi et al., 2011). For example, herbivory attacks induce more extrafloral nectar exudation to recruit more natural enemies (Subedi et al., 2011). In addition, plants with epiphytic habitats are usually poor in resources, and herbivory on epiphytes, such as orchids, is therefore relatively lower than that on nonepiphytes (Winkler, Hülber, Mehltreter, Franco, & Hietz, 2005). When we checked another epiphytic plant family, Bromeliaceae, we were surprised to find that Orchidaceae and Bromeliaceae exhibited the same utilizer ratio (UR).

**Figure 4:**
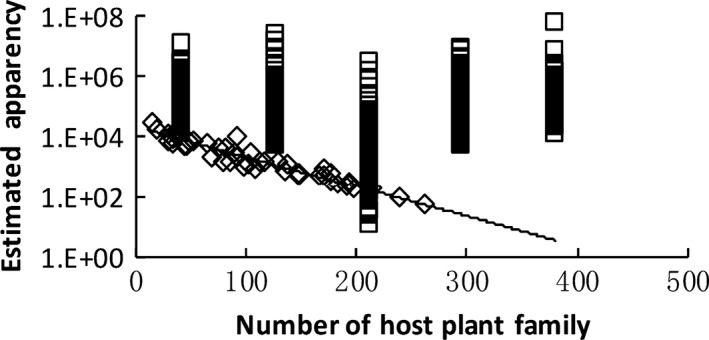
Estimated plant apparency for a utilization probability (UP) = 0.5 (PA_0.5_) decreased with host breadth (log_10_(PA_0.5_) = 4.35 – 0.0010 × HF,* n *=* *46, *r* ^2^ = .92, *p *<* *.00001). □ randomized samples; ◊ actual data

**Figure 5:**
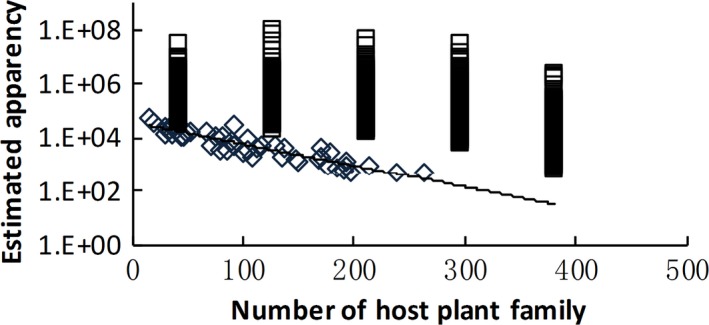
Estimated plant apparency for a utilization probability (UP) = .95 (PA_0.95_) decreased with host breadth (log_10_(PA_0.95_) = 4.59 – 0.0081 × HF,* n *=* *46, *r* ^2^ = .84, *p *<* *.00001). □ randomized samples; 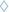 actual data

**Figure 6:**
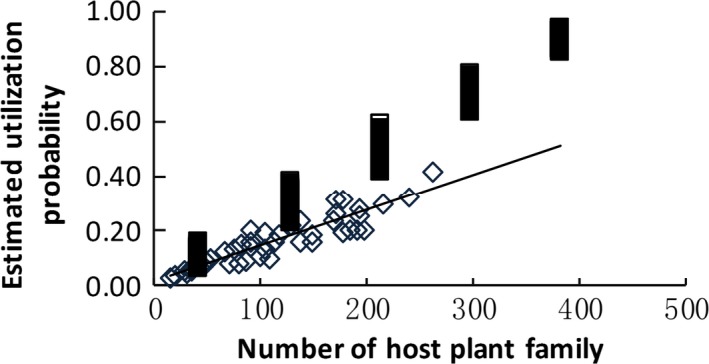
Estimated utilization probability when plant apparency (PA; the species number in a plant family, here) = 0 increased with host breadth (UP_0_ = 0.018 + 0.0013 × HF,* n *=* *46, *r* ^2^ = .85, *p *<* *.00001. □ randomized samples; 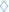 actual data

We then summarized the binomial GLM results for all utilization groups together. The estimated plant apparency values for actual examples at UP* =* 0.5 or 0.95 were generally lower than those for randomly generated samples, indicating strong effects of plant apparency on host plant selection. Expected apparency (PA_0.5_ and PA_0.95_) decreased with host breadth (i.e., the size of the host plant family) according to the actual data (Figures 4 and 5). The *UPs* for most unapparent plant families (PA* *→* *0) were lower than for randomized samples, demonstrating that uncommon plants might escape selection by consumers. The expected UPs (UP_0_) for the actual data increased with host breadth (Figure 6) and the strength of the phylogenetic signal (UP_0_ = 0.13–0.81 × log_10_(*D*), *n *=* *46, *r* ^2^ = .36, *p *=* *.00001) across different consumers. Hence, when the host range is wider (i.e., more host islands are required), unapparent plants (small islands) become more likely to be used.

**Figure 7:**
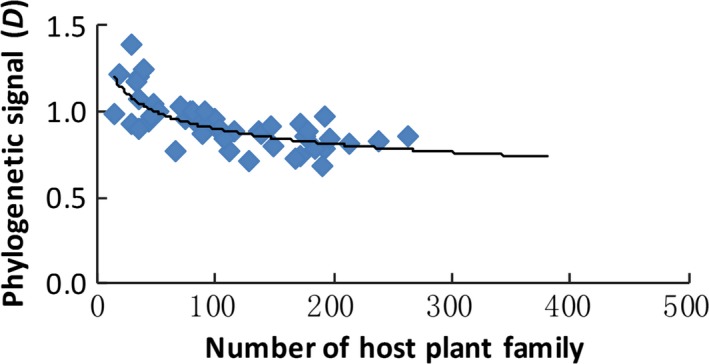
The plant phylogenetic signal decreased with host breadth (*D *=* *1.58–0.34 × log_10_(HF), *n *=* *46, *r* ^2^ = .51, *p *<* *.00001)

Generally, the phylogenetic signals based on utilization presence became stronger (i.e., *D* became smaller) with an increasing host breadth (Figure 7). The phylogenetic signals were significantly different from Brownian motion for all consumers (*D *>* *0.68, *p*_Brownian_ < 0.05; Appendix S8). Furthermore, the phylogenetic signals were remarkably different from random for generalized parasites (pathogens, sap suckers, fruit eaters, plant gallers, and wood borers; *D *<* *0.89, *p*_random_ < 0.1) but not for specialized parasites (leaf miners, leaf rollers, leaf eaters, mutualists, and bark borers; *D *>* *0.89, *p*_random_ > 0.1).

## 4 Discussion

Apparent plants, which are under strong selection pressure from both specialists and generalists, can produce quantitative defenses (tannins and lignin), whereas it is difficult for parasites to specialize toward nonapparent plants, which therefore require only qualitative defenses (alkaloids and terpenoids) against generalists (Strauss et al., 2015). Host plant chemistry may be determined by plant phylogeny (Heidel‐Fischer et al., 2009), where closely related plants share similar biological and chemical defenses and, thus, can be vulnerable to the same types of parasites (Davies & Pedersen, 2008). Alternatively, apparent plants such as trees can facilitate host shifts between phylogenetically distant plants (Heidel‐Fischer et al., 2009).

Similar to nonhuman foragers, humans can behave as specialists or generalists (de Albuquerque, Soldati, & Ramos, 2015). Plant apparency might play a more important role for generalists than for specialists, while the latter are more or less associated with special plant chemicals (Gonçalves et al., 2016; Soldati et al., 2016). Regarding human utilization, the phylogenetic structure differed distinctly from random for wide uses (food, medicines, environmental uses, food additives, materials, and weeds; *D *<* *0.886, *p*_random_ < 0.1), but this was not the case for indirect plant selection by other organisms (forage, vertebrate poisons, invertebrate food, endangered plants, nonvertebrate poisons, hosts of harmful organisms, and bee plants) or for uncommon uses (biomass energy as fuel, gene sources and social uses; *D *>* *0.886, *p*_random_ > 0.1). However, human cultivation and exploitation might increase or decrease the apparency of some particular plant species. For example, it is clear that mono‐cropping plants generally suffer more pest attacks and diseases than their wild relatives.

Consumers at the third trophic level (bodyguard predators and parasitoids) exhibited significant phylogenetic clustering (*p*_random_ = 0.006 and 0.034, respectively); bodyguard predators were more phylogenetically structured than parasitoids (*D *=* *0.767 and 0.851, respectively). Among tritrophic levels, the accumulation of parasitoids is determined by plant commonness, rather than herbivore richness on plants (Nascimento et al., 2014).

The phylogenetic structure of common plant sexual systems, such as hermaphroditism, dioecy, and monoecy (*D *<* *0.99; *p*_random_ < 0.2), was more clumped than that of other, uncommon plant sexual systems (*D *>* *0.99; *p*_*random*_ > 0.4). Plants with different sexual systems not only are associated with different pollination groups (Charlesworth, 1993) but also suffer different herbivory and pathogen pressures (Ashman, 2002; Bertin, Connors, & Kleinman, 2010; Williams, Antonovics, & Rolff, 2011).

At the plant family level, generalized modes of utilization, such as sap sucking, pathogen infection or human medicinal uses, may shift easily from the focal plant family to its relative families because generalized consumers are more likely to utilize new hosts than specialized ones (Forister et al., 2015). Among herbivores, leaf consumers and bark consumers are more specialized than sap consumers and wood consumers. One reason for this difference might be that leaves and bark exhibit more chemical barriers than sap and wood. Highly specialized modes of utilization, such as social uses, are highly species‐specific. The role of one plant species will not be fully replaced by other close relatives of the same genus. For example, the opium poppy (*Papaver somniferum*) is the only species to produce opium in Papaveraceae (Darokar et al., 2014). Once we obtain sufficient global utilization data at the plant genus or species level, we might identify similar patterns. Alternatively, we will be able to test these utilization patterns at lower taxonomic levels on a regional scale when such detailed data are available.

Plants are treated as resources in the ecological apparency hypothesis (Lozano et al., 2014; de Lucena et al., 2007). It would be interesting to extend the apparency hypothesis to animal hosts or abiotic resources. For example, the seven most abundant elements on Earth (iron, oxygen, silicon, magnesium, sulfur, nickel, and calcium) (Morgan & Anders, 1980) but not the 8th most abundant element, aluminum, are also included among the 15 richest elements in the human body.

In summary, our results provide a global illustration of plant–consumer combinations and several general patterns of plant utilization across humans, insects and pathogens. First, plant apparency and plant phylogenetic isolation generally govern plant utilization value. Uncommon and isolated plants suffer fewer parasite attacks. Second, extension of the host breadth utilized helps explain the presence of consumers on unapparent plants. Finally, the phylogenetic clustering structure of host plants is different between common uses and uncommon uses. The strength of such consistent plant utilization patterns across a diverse set of usage types suggests that the persistence and accumulation of consumer diversity and the use value of plant species are determined by similar ecological and evolutionary processes.

## Conflict of Interest

None declared.
