# Supplementary material for: Global pattern of plant utilization across different organisms: Does plant apparency or plant phylogeny matter?
Source: Ecol Evol. 2017 Mar 14;7(8):2535–45. doi: 10.1002/ece3.2882 (PMC5395452; doi:10.1002/ece3.2882)
Supplement: Supplementary file 1 [file ECE3-7-2535-s001.docx]

**SUPPORTING INFORMATION**

Additional Supporting Information may be found in the online version of this article:

Appendix S1 **- Data sources of global plant utilizations**

Appendix S2 – **A Newick file of the full phylogenetic tree of world plant families based on the R2G2_20140601 super tree (Parker et al., 2015) (globalplantfamilies.tree)**

Appendix S3 – **R code to calculate *D* statistic, to fit binomial GLM with logit link and to generate binomial samplings for significance test (PlantUtilization R code.txt)**

Appendix S4 – **ImageJ macro code to batch extract distribution area from maps (ImageJ Macro Code (DistributionArea).txt)**

Appendix S5 – **Prediction of plant utilization probability at global scale when species number in a plant family as plant apparency**

Appendix S6 – **Prediction of plant utilization probability at global scale when distribution area of a plant family as plant apparency**

Appendix S7 – **Prediction of plant utilization probability at global scale when phylogenetic closeness to common plant families as plant phylogenetic closeness**

Appendix S8 – **Phylogenetic signal (*D*) of utilization presence-absence in world plant families**

**Appendix S1 - Data sources of global plant utilizations**

| Utilization group | Consumer type | Main data sources | No. host families in main source | Web of Science hits (Timespan) | No. new host families in WoS |
| --- | --- | --- | --- | --- | --- |
| Agrilus | bark borers | (Jendek & Poláková, 2014) | 75 | 66  (2014-2015) | 0 |
| longicorns | wood borers | (Tavakilian & Chevillotte, 2015) | 193 | updated | 0 |
| Tephritidae | fruit eaters | (Norrbom, 2004) | 50 | 174  (2004-2015) | 8 |
| Cecidomyiidae | gallers | (Gagné, 2004) | 149 | 83  (2004-2015) | 0 |
| food | humans | (National Genetic Resource Program, 2015) | 136 | updated | - |
| food additives | humans | (National Genetic Resource Program, 2015) | 197 | updated | - |
| forages | humans | (National Genetic Resource Program, 2015) | 214 | updated | - |
| medicines | humans | (National Genetic Resource Program, 2015) | 103 | updated | - |
| Invertebrate food | humans | (National Genetic Resource Program, 2015) | 48 | updated | - |
| Environmental uses | humans | (National Genetic Resource Program, 2015) | 15 | updated | - |
| Gene sources | humans | (National Genetic Resource Program, 2015) | 86 | updated | - |
| fuels | humans | (National Genetic Resource Program, 2015) | 53 | updated | - |
| vertebrate poisons | humans | (National Genetic Resource Program, 2015) | 116 | updated | - |
| weeds | humans | (National Genetic Resource Program, 2015) | 138 | updated | - |
| social uses | humans | (National Genetic Resource Program, 2015) | 45 | updated | - |
| CITES endangered plants | humans | (National Genetic Resource Program, 2015) | 46 | updated | - |
| non-vertebrate poisons | humans | (National Genetic Resource Program, 2015) | 29 | updated | - |
| materials | humans | (National Genetic Resource Program, 2015) | 171 | updated | - |
| harmful organism hosts | humans | (National Genetic Resource Program, 2015) | 30 | updated | - |
| bee plants | humans | (National Genetic Resource Program, 2015) | 36 | updated | - |
| extrafloral nectaries | bodyguard predators | (Keeler, 2014) | 110 | 70  (2015) | 1 |
| Cassidinae | leaf eaters | (Borowiec & Swiętojańska, 2015) | 33 | 4  (2015) | 1 |
| Tischeriidae^#^ | leaf miners | (Stonis, 2012), unpubl. data | 19 | 5  (2012-2015) | 0 |
| Leaf-mining Chrysomelidae^#^ | leaf miners | (Santiago-Blay, 2004), unpubl. data | 99 | 36  (2004-2015) | 0 |
| Gracillariidae | leaf miners | (De Prins & De Prins, 2015) | 104 | 21  (2015) | 0 |
| Agromyzidae | leaf miners | (Spencer, 1990) | 136 | 412  (1990-2015) | 6 |
| Tortricidae | leaf rollers | (Brown *et al.*, 2008) | 193 | 225  (2008-2015) | 0 |
| Chalcidoidea | parasitoids | (Noyes, 2015) | 176 | 16  (2015) | 0 |
| nematodes | pathogens | (Ferris, 2015) | 191 | 266  (2015) | 0 |
| virus | pathogens | (Brunt *et al.*, 1996) | 109 | - | - |
| fungi | pathogens | (Farr & Rossman, 2006) | 258 | 224  (2006-2015) | 2 |
| dioecy | pollinators | (Renner & Ricklefs, 1995; Ashman *et al.*, 2014) | 169 | 435  (2014-2015) | 1 |
| gynomonoecy | pollinators | (Ashman *et al.*, 2014; Mamut & Tan, 2014) | 34 | 15  (2014-2015) | 1 |
| andromonoecy | pollinators | (Miller & Diggle, 2003; Ashman *et al.*, 2014), Diggle P (*Pers Comm*) | 24 | 198  (2003-2015) | 8 |
| monoecy | pollinators | (Yampolsky & Yampolsky, 1922; Ashman *et al.*, 2014) | 75 | 361  (1926-2015) | 8 |
| hermaphrodite | pollinators | (Yampolsky & Yampolsky, 1922; Ashman *et al.*, 2014) | 154 | 228  (1926-2015) | 12 |
| gynodioecy | pollinators | (Ashman *et al.*, 2014; Dufay *et al.*, 2014) | 75 | 402  (1926-2015) | 2 |
| polygamodioecy^&^ | pollinators | (Yampolsky & Yampolsky, 1922; Ashman *et al.*, 2014) | 68 | 96  (1926-2015) | 1 |
| polygamomonoecy^$^ | pollinators | (Yampolsky & Yampolsky, 1922; Ashman *et al.*, 2014) | 67 | 188  (1926-2015) | 7 |
| androdioecy | pollinators | (Yampolsky & Yampolsky, 1922; Ashman *et al.*, 2014) | 28 | 367  (1926-2015) | 3 |
| aphids | sap suckers | (Blackman & Eastop, 2014) | 239 | 455  (2015) | 0 |
| whiteflies | sap suckers | (Ouvrard & Martin, 2015) | 88 | 71  (2015) | 2 |
| plant hoppers | sap suckers | (Bourgoin, 2015) | 129 | updated | - |
| psyllids | sap suckers | (Ouvrard, 2015) | 167 | updated | - |
| spider mites | sap suckers | (Migeon & Dorkeld, 2015) | 184 | 51  (2015) | 0 |
| arbuscular mycorrhizal fungi | mutualists | (Öpik *et al.*, 2010) | 75 | 225  (2010-2015) | 8 |

Additional host plant families for Tischeriidae and leaf-mining Chrysomelidae were added according to our field collections in China. # the website is inaccessible now. & polygamodioecious in “Tree of Sex: A database of sexual systems” includes polygamodioecious and trioecious in “Distribution of sex forms in the phanerogamic flora”. $ polygamomonoecious in “Tree of Sex: A database of sexual systems” = polygamous in “Distribution of sex forms in the phanerogamic flora”.

**REFERENCES for Appendix S1**

Ashman, T.-L., Bachtrog, D., Blackmon, H., Goldberg, E.E., Hahn, M.W., Kirkpatrick, M., Kitano, J., Mank, J.E., Mayrose, I., Ming, R., Otto, S.P., Peichel, C.L., Pennell, M.W., Perrin, N., Ross, L., Valenzuela, N. & Vamosi, J.C. (2014) Tree of Sex: A database of sexual systems. *Scientific Data*, **1**, 140015.

Blackman, R.L. & Eastop, V.F. (2014) Aphids on the World’s Plants: An online identification and information guide.

Borowiec, L. & Swiętojańska, J. (2015) Cassidinae of the world-an interactive manual (Coleoptera: Chrysomelidae).

Bourgoin, T. (2015) FLOW (Fulgoromorpha Lists on The Web): a world knowledge base dedicated to Fulgoromorpha.

Brown, J.W., Robinson, G. & Powell, J.A. (2008) Food plant database of the leafrollers of the world (Lepidoptera: Tortricidae) (Version 1.0).

Brunt, A.A., Crabtree, K., Dallwitz, M.J., Gibbs, A.J., Watson, L. & Zurcher, E.J. (1996) Plant viruses online: descriptions and lists from the VIDE database.

Dufay, M., Champelovier, P., Kafer, J., Henry, J.P., Mousset, S. & Marais, G.A.B. (2014) An angiosperm-wide analysis of the gynodioecy-dioecy pathway. *Annals of Botany*, **114**, 539–548.

Farr, D.F. & Rossman, A.Y. (2006) Fungal Databases, Systematic Mycology and Microbiology Laboratory, ARS, USDA.

Ferris, H. (2015) Nemabase- A Database of the Host Status of Plants to Nematodes.

Gagné, R.J. (2004) A Catalog of the Cecidomyiidae (Diptera) of the World. *Memoirs of the Entomological Society of Washington*, **25**, 1–544.

Jendek, E. & Poláková, J. (2014) *Host plants of world Agrilus (Coleoptera, Buprestidae): A critical review*, Springer.

Keeler, K.H. (2014) World list of plants with extrafloral nectaries.

Mamut, J. & Tan, D.Y. (2014) Gynomonoecy in angiosperms: phylogeny, sex expression and evolutionary significance. *Chinese Journal of Plant Ecology*, **38**, 76–90.

Migeon, A. & Dorkeld, F. (2015) Spider Mites Web: a comprehensive database for the Tetranychidae.

Miller, J.S. & Diggle, P.K. (2003) Diversification of andromonoecy in *Solanum* section *Lasiocarpa* (Solanaceae): The roles of phenotypic plasticity and architecture. *American Journal of Botany*, **90**, 707–715.

National Genetic Resource Program (2015) Germplasm Resources Information Network-(GRIN).

Norrbom, A. (2004) Fruit Fly Host Plant Database.

Noyes, J.S. (2015) Universal Chalcidoidea Database.

Öpik, M., Vanatoa, A., Vanatoa, E., Moora, M., Davison, J., Kalwij, J.M., Reier, Ü. & Zobel, M. (2010) The online database Maarj*AM* reveals global and ecosystemic distribution patterns in arbuscular mycorrhizal fungi (Glomeromycota). *New Phytologist*, **188**, 223–241.

Ouvrard, D. (2015) Psyl’list - The World Psylloidea Database.

Ouvrard, D. & Martin, J.H. (2015) The White-files - Taxonomic checklist of the world’s whiteflies (Insecta: Hemiptera: Aleyrodidae).

De Prins, J. & De Prins, W. (2015) Global taxonomic database of Gracillariidae (Lepidoptera).

Renner, S.S. & Ricklefs, R.E. (1995) Dioecy and its correlates in the flowering plants. *American Journal of Botany*, **82**, 596–606.

Santiago-Blay, J.A. (2004) *Leaf-mining chrysomelids*, (ed. by P. Jolivet), J.A. Santiago-Blay), and M. Schmitt) Academic Publishing, The Hague.

Spencer, K.A. (1990) *Host Specialization in the World Agromyzidae (Diptera)*, Kluwer Academic Publishers, Dordrecht, The Netherlands.

Stonis, J.R. (2012) Global diversity & phylogeny of Tischeriidae.

Tavakilian, G. & Chevillotte, H. (2015) Base de données Titan sur les Cerambycidés ou Longicornes.

Yampolsky, C. & Yampolsky, H. (1922) Distribution of sex forms in the phanerogamic flora. *Bibliotecha Genetica*, **3**, 1–62.

Appendix S2 – **A Newick file of the full phylogenetic tree of world plant families based on the R2G2_20140601 super tree (Parker et al., 2015) (globalplantfamilies.tree)**

((lycopodiaceae:410.0,(selaginellaceae:205.0,isoetaceae:205.0)n00001:205.0)lycophytes:36.0,(((((((((((((((((((((polypodiaceae:19.285719,davalliaceae:19.285719)n00002:19.285719,oleandraceae:38.571438)n00003:19.285717,tectariaceae:57.857155)n00004:19.28572,nephrolepidaceae:77.142876)n00005:19.28572,lomariopsidaceae:96.4286)n00006:19.285713,dryopteridaceae:115.71431)n00007:19.285719,hypodematiaceae:135.00003)eupolypodsI:19.285706,(((((((athyriaceae:34.28572,(onocleaceae:17.14286,blechnaceae:17.14286)n00008:17.14286)n00009:17.14286,woodsiaceae:51.42858)n00010:17.14286,rhachidosoraceae:68.57144)n00011:17.14286,thelypteridaceae:85.7143)n00012:17.14286,aspleniaceae:102.85716)n00013:17.14286,cystopteridaceae:120.00002)n00014:17.14286,diplaziopsidaceae:137.14288)eupolypodsII:17.142853)n00015:17.142853,dennstaedtiaceae:171.42859)n00016:17.142853,pteridaceae:188.57144)n00017:17.142853,(saccolomataceae:154.28572,(cystodiaceae:102.85715,(lonchitidaceae:51.428574,lindsaeaceae:51.428574)n00018:51.428574)n00019:51.428574)n00020:51.428574)n00021:17.142853,((((culcitaceae:44.57143,plagiogyriaceae:44.57143)n00022:44.57143,loxsomataceae:89.14286)n00023:44.571434,thyrsopteridaceae:133.7143)n00024:44.571426,(cyatheaceae:133.7143,(cibotiaceae:89.14286,(metaxyaceae:44.57143,dicksoniaceae:44.57143)n00025:44.57143)n00026:44.571434)n00028:44.571426)n00029:44.571426)n00030:17.142868,(salviniaceae:120.00001,marsileaceae:120.00001)n00031:120.00001)n00032:17.142868,(lygodiaceae:171.42859,(anemiaceae:85.714294,schizaeaceae:85.714294)n00033:85.714294)n00034:85.714294)n00035:17.142822,((matoniaceae:91.428566,dipteridaceae:91.428566)n00036:91.428566,gleicheniaceae:182.85713)n00037:91.42857)n00038:17.142883,hymenophyllaceae:291.4286)n00039:17.142822,osmundaceae:308.5714)n00040:17.142883,equisetaceae:325.7143)n00041:17.142853,marattiaceae:342.85715)n00042:17.142855,(ophioglossaceae:180.0,psilotaceae:180.0)n00043:180.0)monilophytes:43.0,((ginkgoaceae:319.0,(cycadaceae:159.5,zamiaceae:159.5)cycadales:159.5,(((araucariaceae:133.33333,podocarpaceae:133.33333)n00063:133.33333,(sciadopityaceae:223.33333,taxaceae:223.33333,cupressaceae:223.33333)n00077:43.33333)n00078:43.333332,pinaceae:310.0)crownconifers:9.0)gymnosperms:6.0,(((gnetaceae:135.0,welwitschiaceae:135.0)n00079:135.0,ephedraceae:270.0)gnetales:27.5,(((((((((((((((((((((((caprifoliaceae:81.0,adoxaceae:81.0)dipsacales:3.604164,paracryphiaceae:84.604164)n00080:3.604164,((((((apiaceae:11.5,myodocarpaceae:11.5)n00081:11.5,araliaceae:23.0)n00082:11.5,pittosporaceae:34.5)n00083:11.5,griseliniaceae:46.0)n00084:11.5,torricelliaceae:57.5)n00085:11.5,pennantiaceae:69.0)apiales:19.208328)n00086:3.604164,(bruniaceae:45.90625,columelliaceae:45.90625)bruniales:45.906242)n00087:3.604164,escalloniaceae:95.41666,(((((((calyceraceae:48.857143,asteraceae:48.857143)n00159:6.857143,goodeniaceae:55.714287)n00160:6.85714,menyanthaceae:62.571426)n00161:6.857147,stylidiaceae:69.42857)n00162:6.85714,(alseuosmiaceae:50.857143,(phellinaceae:25.428572,argophyllaceae:25.428572)n00163:25.428572)n00164:25.42857)n00165:6.85714,pentaphragmataceae:83.14285)n00166:6.857143,(campanulaceae:45.0,rousseaceae:45.0)n00167:45.0)asterales:5.416656)n00168:5.416656,(((phyllonomaceae:32.333332,helwingiaceae:32.333332)n00202:32.333332,aquifoliaceae:64.666664)n00169:32.333332,(stemonuraceae:48.5,cardiopteridaceae:48.5)n00170:48.5)aquifoliales:3.833313)campanulids:3.833344,((((((((((((((((((paulowniaceae:4.2,orobanchaceae:4.2)n00171:4.2,phrymaceae:8.4)n00203:4.2,mazaceae:12.599999)n00204:4.2,lamiaceae:16.8)n00172:4.200001,((thomandersiaceae:7.0,verbenaceae:7.0)n00173:7.0,(schlegeliaceae:7.0,lentibulariaceae:7.0)n00174:7.0)n00175:7.0)n00176:4.199999,bignoniaceae:25.199999)n00177:4.199999,(pedaliaceae:14.699999,martyniaceae:14.699999,acanthaceae:14.699999)n00178:14.699999)n00179:4.200001,(byblidaceae:16.8,linderniaceae:16.8)n00180:16.8)n00181:4.200001,stilbaceae:37.8)n00182:4.200001,scrophulariaceae:42.0)n00183:4.199997,plantaginaceae:46.199997)n00184:4.200001,(calceolariaceae:25.199999,gesneriaceae:25.199999)n00185:25.199999)n00186:4.200001,tetrachondraceae:54.6)n00187:4.199997,(carlemanniaceae:29.399998,oleaceae:29.399998)n00188:29.399998)n00189:4.2,plocospermataceae:63.0)lamiales:21.944435,((convolvulaceae:59.5,solanaceae:59.5)n00252:18.5,((sphenocleaceae:26.0,hydroleaceae:26.0)n00253:26.0,montiniaceae:52.0)n00254:26.0)solanales:6.944435)n00255:6.944443,((((apocynaceae:17.75,gelsemiaceae:17.75)n00256:17.75,loganiaceae:35.5)n00257:17.75,gentianaceae:53.25)n00258:17.75,rubiaceae:71.0)gentianales:20.888878,boraginaceae:91.88888)n00259:6.944438,(garryaceae:93.0,eucommiaceae:93.0)garryales:5.833313,(oncothecaceae:81.91666,metteniusaceae:81.91666,icacinaceae:81.91666)unplacedlamiids:16.916656)lamiids:5.833344)euasterids:6.166672,(((((((cyrillaceae:57.142857,ericaceae:57.142857)n00264:7.142857,clethraceae:64.28571)n00265:7.14286,((actinidiaceae:23.809525,roridulaceae:23.809525)n00266:23.809525,sarraceniaceae:47.61905)n00267:23.809525)n00268:7.142853,(mitrastemonaceae:58.92857,theaceae:58.92857,((styracaceae:19.642857,diapensiaceae:19.642857)n00269:19.642857,symplocaceae:39.285713)n00270:19.642857)n00271:19.642857)n00272:7.14286,(((primulaceae:21.428572,ebenaceae:21.428572)n01207:21.428572,sapotaceae:42.857143)n00273:21.42857,(sladeniaceae:32.142857,pentaphylacaceae:32.142857)n00274:32.142857)n00275:21.428574)n00276:7.14286,(lecythidaceae:61.904766,(polemoniaceae:30.952383,fouquieriaceae:30.952383)n00277:30.952383)n00278:30.952381)n00279:7.142857,((tetrameristaceae:33.333332,marcgraviaceae:33.333332)n01208:33.333332,balsaminaceae:66.666664)n00280:33.333332)ericales:10.833328)n00281:6.166672,(((loasaceae:37.875,hydrangeaceae:37.875)n00282:37.875,(cornaceae:50.5,(curtisiaceae:25.25,grubbiaceae:25.25)n00283:25.25)n00284:25.25)n00285:25.25,hydrostachyaceae:101.0)cornales:16.0)asterids:2.0,(((((((((((((((portulacaceae:4.259741,cactaceae:4.259741)n00286:4.259741,anacampserotaceae:8.519482)n00287:4.259741,talinaceae:12.779222)n00288:4.259741,(halophytaceae:8.519482,didiereaceae:8.519482,basellaceae:8.519482)n00289:8.519482)n00290:4.259741,montiaceae:21.298704)n00291:4.259741,molluginaceae:25.558445)n00292:4.259741,((((phytolaccaceae:5.963637,gisekiaceae:5.963637,sarcobataceae:5.963637,nyctaginaceae:5.963637)n00293:5.963637,aizoaceae:11.927275)n01209:5.963636,barbeuiaceae:17.890911)n00294:5.963638,lophiocarpaceae:23.85455)n00295:5.963636)n00296:4.259741,limeaceae:34.077927)n00297:4.259743,stegnospermataceae:38.33767)n00298:4.259739,((caryophyllaceae:14.199136,achatocarpaceae:14.199136)n00299:14.199136,amaranthaceae:28.398272)n00300:14.199137)n00301:4.259741,microteaceae:46.857147)core_caryophyllales:9.285717,(asteropeiaceae:28.071432,physenaceae:28.071432)n01210:28.071432)n00302:9.285717,simmondsiaceae:65.42858)n00303:9.285716,rhabdodendraceae:74.714294)rhabdoClade:9.285706,(((((ancistrocladaceae:14.0,dioncophyllaceae:14.0)n00304:14.0,drosophyllaceae:28.0)n00305:14.0,nepenthaceae:42.0)n00306:14.0,droseraceae:56.0)n00307:14.0,((plumbaginaceae:23.333334,polygonaceae:23.333334)n00308:23.333334,(frankeniaceae:23.333334,tamaricaceae:23.333334)n00309:23.333334)n00310:23.333332)n00311:14.0)caryophyllales:35.0)n00312:2.0,(((((((santalaceae:12.9375,opiliaceae:12.9375)n00313:12.9375,((schoepfiaceae:8.625,misodendraceae:8.625)n01211:8.625,loranthaceae:17.25)n00314:8.625)n00315:8.625,octoknemaceae:34.5)n00316:8.625,(ximeniaceae:21.5625,aptandraceae:21.5625,olacaceae:21.5625)n01212:21.5625)n00317:8.625,coulaceae:51.75)n00318:8.625,(erythropalaceae:30.1875,strombosiaceae:30.1875)n00319:30.1875)n00320:8.625,balanophoraceae:69.0)santalales:52.0)n00321:2.0,(aextoxicaceae:61.5,berberidopsidaceae:61.5)berberidopsidales:61.5)n00322:2.0,((((((((((((malvaceae:40.800003,(sphaerosepalaceae:27.2,bixaceae:27.2,(cistaceae:13.6,sarcolaenaceae:13.6,dipterocarpaceae:13.6)n00323:13.6,(cytinaceae:13.6,muntingiaceae:13.6)n00324:13.6)n00325:13.600002)n00326:13.599998,thymelaeaceae:54.4)n00327:13.6,neuradaceae:68.0)malvales:13.666664,(((((((((brassicaceae:8.777778,cleomaceae:8.777778)n01213:8.777778,capparaceae:17.555555)n00328:8.777777,(pentadiplandraceae:17.555555,(resedaceae:8.777778,gyrostemonaceae:8.777778)n00329:8.777778,tovariaceae:17.555555)n00330:8.777777)n00331:8.777779,emblingiaceae:35.11111)n00332:8.777779,((bataceae:14.62963,salvadoraceae:14.62963)n00333:14.62963,koeberliniaceae:29.25926)n00334:14.629629)n00335:8.777775,limnanthaceae:52.666664)n00336:8.777779,setchellanthaceae:61.444443)n00337:8.777779,(moringaceae:35.11111,caricaceae:35.11111)n00338:35.11111)n00339:8.777778,(akaniaceae:39.5,tropaeolaceae:39.5)n00340:39.5)brassicales:2.666664)n00341:2.666672,(gerrardinaceae:42.166656,petenaeaceae:42.166656,dipentodontaceae:42.166656,tapisciaceae:42.166656)huerteales:42.16668)n00342:2.666664,(((((rutaceae:43.0,(simaroubaceae:36.5,meliaceae:36.5)n00358:6.5)n00359:6.5,sapindaceae:49.5)n00360:6.5,((anacardiaceae:51.0,burseraceae:51.0)bursa2anaca:2.5,kirkiaceae:53.5)n00361:2.5)n00362:2.5,nitrariaceae:58.5)n01214:2.5,biebersteiniaceae:61.0)sapindales:26.0)n00363:2.666664,picramniaceae:89.666664)n00364:2.666672,((((strasburgeriaceae:15.5,ixerbaceae:15.5)n01215:15.5,geissolomataceae:31.0)n00365:15.5,aphloiaceae:46.5)n00366:15.5,(((stachyuraceae:15.5,crossosomataceae:15.5)n00367:15.5,guamatelaceae:31.0)n00368:15.5,staphyleaceae:46.5)n00369:15.5)crossosomatales:30.333336)n00370:2.666667,(((((((penaeaceae:14.666667,alzateaceae:14.666667)n00371:14.666667,crypteroniaceae:29.333334)n00372:14.666666,melastomataceae:44.0)n00373:14.666668,(myrtaceae:29.333334,vochysiaceae:29.333334)n00374:29.333334)n00375:14.666668,(onagraceae:36.666668,lythraceae:36.666668)n00376:36.666668)n00377:14.666667,combretaceae:88.0)myrtales:5.5,(geraniaceae:92.0,melianthaceae:92.0,vivianiaceae:92.0)geraniales:1.5)n00378:1.5)malvids:1.5,((((((((((begoniaceae:13.0,datiscaceae:13.0)n00379:13.0,tetramelaceae:26.0)n01216:13.0,cucurbitaceae:39.0)n00380:13.0,((corynocarpaceae:17.333334,coriariaceae:17.333334)n00381:17.333334,apodanthaceae:34.666668)n00382:17.333332)n00383:13.0,anisophylleaceae:65.0)cucurbitales:7.662498,((((casuarinaceae:23.75,(ticodendraceae:11.875,betulaceae:11.875)n00384:11.875)n00385:11.875,((juglandaceae:11.875,rhoipteleaceae:11.875)n00386:11.875,myricaceae:23.75)n00387:11.875)n00388:11.875,fagaceae:47.5)n00397:13.5,nothofagaceae:61.0)fagales:11.662498)n00398:7.662498,(((((urticaceae:33.6,moraceae:33.6)n01218:10.600002,cannabaceae:44.2)n00429:10.600002,ulmaceae:54.800003)n00430:10.599998,((dirachmaceae:21.800001,rhamnaceae:21.800001,elaeagnaceae:21.800001)n00431:21.800001,barbeyaceae:43.600002)n00432:21.8)n00433:10.6,rosaceae:29.0)rosales:4.324997)n00434:4.324997,(((surianaceae:33.75,polygalaceae:33.75)n00435:33.75,fabaceae:67.50001)n00462:11.5,quillajaceae:79.0)fabales:5.649994)n00463:5.650002,(((((goupiaceae:44.833332,(violaceae:22.416666,passifloraceae:22.416666)n00464:22.416666,(lacistemataceae:22.416666,salicaceae:22.416666)n00465:22.416666)n00466:22.416666,achariaceae:67.25)parietalClade:13.75,((rafflesiaceae:27.0,euphorbiaceae:27.0)n00467:27.0,peraceae:54.0)n00468:27.0,(picrodendraceae:59.5,phyllanthaceae:59.5)phyllanthoids:21.5,(((hypericaceae:20.25,podostemaceae:20.25)n00469:20.25,calophyllaceae:40.5)n00470:20.25,(bonnetiaceae:30.375,clusiaceae:30.375)n00471:30.375)clusioids:20.25,ochnaceae:81.0,(((trigoniaceae:20.25,dichapetalaceae:20.25)n00472:20.25,(chrysobalanaceae:20.25,euphroniaceae:20.25)n00473:20.25)n00474:20.25,balanopaceae:60.75)n00475:20.25,(ctenolophonaceae:54.0,(erythroxylaceae:27.0,rhizophoraceae:27.0)n00476:27.0)n00477:27.0,(elatinaceae:40.5,malpighiaceae:40.5)n00478:40.5,linaceae:81.0,humiriaceae:81.0,ixonanthaceae:81.0,pandaceae:81.0,caryocaraceae:81.0,centroplacaceae:81.0,irvingiaceae:81.0,(lophopyxidaceae:40.5,putranjivaceae:40.5)n00479:40.5)malpighiales:3.099998,((((elaeocarpaceae:30.8,(brunelliaceae:15.4,cephalotaceae:15.4)n01514:15.4)n01220:15.399998,cunoniaceae:46.199997)n00480:15.400002,(connaraceae:30.8,oxalidaceae:30.8)n00481:30.8)n01221:15.4,huaceae:77.0)oxalidales:7.099998)n00482:3.099998,(celastraceae:42.0,lepidobotryaceae:42.0)celastrales:45.199997)n00483:3.099998)n00484:3.099998,(zygophyllaceae:70.0,krameriaceae:70.0)zygophyllales:23.399994)fabids:3.100006)eurosids:1.5,vitaceae:98.0)rosids:20.0,(((((saxifragaceae:26.428572,grossulariaceae:26.428572)n01222:26.428572,iteaceae:52.857143)n00485:26.42857,((((haloragaceae:15.857142,penthoraceae:15.857142)n00486:15.857142,tetracarpaeaceae:31.714285)n01223:15.857141,aphanopetalaceae:47.571426)n00487:15.857143,crassulaceae:63.42857)n00488:15.857143)n00489:15.85714,((((cercidiphyllaceae:19.02857,daphniphyllaceae:19.02857)n00490:19.02857,hamamelidaceae:38.05714)n00491:19.028568,altingiaceae:57.08571)n00492:19.028572,paeoniaceae:76.11428)n00493:19.028572)n00494:15.857142,peridiscaceae:111.0,vahliaceae:111.0,cynomoriaceae:111.0)saxifragales:7.0)n00495:7.0,dilleniaceae:125.0)n00496:2.0,(gunneraceae:63.5,myrothamnaceae:63.5)gunnerales:63.5)core_eudicots:7.0,(buxaceae:115.5,haptanthaceae:115.5)buxales:18.5,trochodendraceae:134.0)n00497:7.0,((platanaceae:67.5,proteaceae:67.5)n00498:67.5,nelumbonaceae:135.0)proteales:6.0,sabiaceae:141.0)n00499:6.0,(((((ranunculaceae:28.0,berberidaceae:28.0)n00500:28.0,menispermaceae:56.0)n00501:28.0,(circaeasteraceae:42.0,lardizabalaceae:42.0)n00502:42.0)n00503:28.0,papaveraceae:112.0)n01224:28.0,eupteleaceae:140.0)ranunculales:7.0)eudicots:3.814819,ceratophyllaceae:150.81482)n00504:3.814804,((((((((((((strelitziaceae:15.5,lowiaceae:15.5)n00505:15.5,musaceae:31.0)n00506:15.5,((marantaceae:15.5,cannaceae:15.5)n00507:15.5,(zingiberaceae:15.5,costaceae:15.5)n00508:15.5)n00509:15.5)n00510:15.5,heliconiaceae:62.0)zingiberales:18.411102,((commelinaceae:34.0,hanguanaceae:34.0)n00511:34.0,(philydraceae:45.333332,(pontederiaceae:22.666666,haemodoraceae:22.666666)n00512:22.666666)n00513:22.666666)commelinales:12.411102)n00514:12.411102,(((((((joinvilleaceae:42.201588,ecdeiocoleaceae:42.201588)n01327:42.201588,poaceae:84.403175)n01328:1.403168,flagellariaceae:85.80634)n01329:1.403175,((restionaceae:29.06984,centrolepidaceae:29.06984)n01330:29.06984,anarthriaceae:58.13968)n01331:29.06984)n01332:1.403168,((((cyperaceae:17.722538,juncaceae:17.722538)n01333:17.722538,thurniaceae:35.445076)n01334:17.722538,mayacaceae:53.167614)n01335:17.722538,(eriocaulaceae:35.445076,xyridaceae:35.445076)n01336:35.445076)n01337:17.722534)n01338:1.403175,rapateaceae:90.01586)n01339:1.403175,(typhaceae:45.70952,bromeliaceae:45.70952)n01340:45.70952)poales:1.403172,dasypogonaceae:92.822205,arecaceae:92.822205)commelinids:19.822243,((((((((asparagaceae:13.375,amaryllidaceae:13.375)n01510:13.375,xanthorrhoeaceae:26.75)n01341:13.375,xeronemataceae:40.125)n01342:13.375,iridaceae:53.5)n01343:13.375,doryanthaceae:66.875)n01344:13.375,(tecophilaeaceae:40.125,ixioliriaceae:40.125)n01345:40.125)n01346:13.375,((((hypoxidaceae:18.725,lanariaceae:18.725)n01347:18.725,asteliaceae:37.45)n01348:18.725002,blandfordiaceae:56.175003)n01349:18.724998,boryaceae:74.9)n01350:18.724998)n01351:13.375,orchidaceae:107.0)asparagales:5.644447)n01352:5.644447,(((melanthiaceae:57.600002,((smilacaceae:19.2,liliaceae:19.2)n01353:19.2,(rhipogonaceae:19.2,philesiaceae:19.2)n01354:19.2)n01355:19.2,((colchicaceae:19.2,alstroemeriaceae:19.2)n01356:19.2,petermanniaceae:38.4)n01357:19.2)n01358:19.2,campynemataceae:76.8)n01359:19.2,corsiaceae:96.0)liliales:22.288895)n01360:5.64444,((((cyclanthaceae:34.699993,pandanaceae:34.699993)n01361:34.699993,stemonaceae:69.39999)n01362:34.699993,triuridaceae:104.099976,velloziaceae:104.099976)pandanales:9.916679,(burmanniaceae:95.0,dioscoreaceae:95.0,nartheciaceae:95.0)dioscoreales:19.016655)n01363:9.916679)n01364:5.644455,petrosaviaceae:129.57779)n01365:5.64444,((((((((((cymodoceaceae:22.545454,(najadaceae:11.272727,zosteraceae:11.272727)n01366:11.272727)n01367:11.272726,posidoniaceae:33.81818)n01368:11.272728,((potamogetonaceae:15.030303,zannichelliaceae:15.030303)n01369:15.030303,ruppiaceae:30.060606)n01370:15.030302)n01371:11.272728,(lilaeaceae:28.181818,juncaginaceae:28.181818)n01372:28.181818)n01373:11.272724,scheuchzeriaceae:67.63636)n01374:11.272728,aponogetonaceae:78.90909)n01375:11.272728,(hydrocharitaceae:67.63636,((alismataceae:22.545454,limnocharitaceae:22.545454)n01376:22.545454,butomaceae:45.090908)n01377:22.545452)n01378:22.545456)n01379:11.272728,lemnaceae:101.454544)n01380:11.272728,tofieldiaceae:112.72727)n01381:11.272727,araceae:124.0)alismatales:11.222229)n01489:11.222229,acoraceae:146.44446)monocots:8.185165)n01490:3.814819,(((((((lauraceae:27.75,monimiaceae:27.75)n01491:27.75,hernandiaceae:55.5)n01492:27.75,((gomortegaceae:27.75,atherospermataceae:27.75)n01493:27.75,siparunaceae:55.5)n01494:27.75)n01495:27.75,calycanthaceae:111.0)laurales:19.25,(((annonaceae:37.666668,eupomatiaceae:37.666668)n01496:37.666668,(himantandraceae:37.666668,degeneriaceae:37.666668)n01497:37.666668,magnoliaceae:75.333336)n01498:37.666668,myristicaceae:113.0)magnoliales:17.25)n01499:17.25,(((saururaceae:66.0,piperaceae:66.0)n01500:66.0,(lactoridaceae:66.0,hydnoraceae:66.0,aristolochiaceae:66.0)n01501:66.0)piperales:7.75,(winteraceae:99.0,canellaceae:99.0)canellales:40.75)n01502:7.75)magnoliids:5.472229,chloranthaceae:152.97223)n01503:5.472214)n01504:5.472214,(austrobaileyaceae:84.16666,schisandraceae:84.16666,trimeniaceae:84.16666)austrobaileyales:79.75)n01505:5.472229,((nymphaeaceae:79.888885,cabombaceae:79.888885)n01506:79.888885,hydatellaceae:159.77777)nymphaeales:9.611115)n01507:9.611115,amborellaceae:179.0)angiosperms:118.5)n01508:27.5)seedplants:78.0)n01509:43.0)vascularplants;

Appendix S3 – **R code to calculate *D* statistic, to fit binomial GLM with logit link and to generate binomial samplings for significance test (PlantUtilizatiion R code.txt)**

#load required R packages

require(caper)

require(picante)

require(nls2)

require(geiger)

#set work directory

setwd("E:/your working directory")

#read presence/absence data of plant utilization

hf <- read.table("host0-1.txt", header = T) #data source: 'Fig. 1&2' sheet in 'DataforFig1-3.xls'

#read the phylogenetic megatree of all plant families

hft <- read.tree("globalplantfamilies.tree")

#calculate the phylogenetic D statistic for Tischeriidae, similar code for other utilizers

D <- phylo.d(hf,hft,hsfa,tisc)

print(D)

#initialize arrays for binomial GLM fitting

x5 <- NULL

x95 <- NULL

y0 <- NULL

aic <- NULL

a <- NULL

b <- NULL

#loop to fit all utilization group

for(i in 8:53){

y <- unlist(hf[i]) #presence/absence of utilization in each group

x <- hf$hssn #species number, you can replace hssn with land, hssn or pdmin

#binomial GLM fitting of utilization presence with plant apparency or phylogenetic closeness

fm <- glm(y~x,family=binomial(link="logit"))

#plots and binomial GLM parameters

plot(y~x)

aic <- c(aic,AIC(fm))

coefs <- coef(fm)

a <- c(a, coefs[2])

b <- c(b, coefs[1])

plot(y~x)

curve((exp(coefs[2]*x+coefs[1])/(1+exp(coefs[2]*x+coefs[1]))),col="red", lwd=2, add=T)

#estimated plant apparency or phylogenetic closeness when utilization probability = 0.5

x5 <- c(x5,(log(0.5/(1-0.5))-coefs[1])/coefs[2])

#estimated plant apparency or phylogenetic closeness when utilization probability = 0.95

x95 <- c(x95,(log(0.95/(1-0.95))-coefs[1])/coefs[2])

#estimated utilization probability when plant apparency = 0 or phylogenetic closeness = 0

y0 <- c(y0,(exp(coefs[2]*0+coefs[1])/(1+exp(coefs[2]*0+coefs[1]))))

}

write.table(data.frame(aic,x5,x95,y0,a,b), "hssn.txt") #output binomial fitting data

#data frame for the generation of binomial samplings

pf <- data.frame(hf$hsfa,hf$hssn,hf$hsgn,hf$land,hf$pdmin)

#array initialization

pfr1 <- pf

pfr3 <- pf

pfr5 <- pf

pfr7 <- pf

pfr9 <- pf

#generate 1000 binomial samples for 423 plant families

for (i in 1:1000){

pfr1 <- data.frame(pfr1,rbinom(423,1,0.1))

pfr3 <- data.frame(pfr3,rbinom(423,1,0.3))

pfr5 <- data.frame(pfr5,rbinom(423,1,0.5))

pfr7 <- data.frame(pfr7,rbinom(423,1,0.7))

pfr9 <- data.frame(pfr9,rbinom(423,1,0.9))

}

#output random generation data

write.table(pfr1, "pfr1.txt")

write.table(pfr3, "pfr3.txt")

write.table(pfr5, "pfr5.txt")

write.table(pfr7, "pfr7.txt")

write.table(pfr9, "pfr9.txt")

#array initialization

ra <- NULL

rb <- NULL

rx5 <- NULL

rx95 <- NULL

ry0 <- NULL

for (i in 5:1004){

#random generation data for pfr1, which can be replaced by prf3, prf5, prf7, or prf9

y <- unlist(pfr1[i])

x <- unlist(pfr1$hf.hssn) #species number, you can replace hssn with land, hssn or pdmin

#binomial GLM fitting of utilization presence with plant apparency or phylogenetic closeness

fm <- glm(y~x,family=binomial(link="logit"))

coefs <- coef(fm)

ra <- c(ra, coefs[2])

rb <- c(rb, coefs[1])

#estimated plant apparency or phylogenetic closeness when utilization probability = 0.5

rx5 <- c(rx5,(log(0.5/(1-0.5))-coefs[1])/coefs[2])

#estimated plant apparency or phylogenetic closeness when utilization probability = 0.95

rx95 <- c(rx95,(log(0.95/(1-0.95))-coefs[1])/coefs[2])

#estimated utilization probability when plant apparency = 0 or phylogenetic closeness = 0

ry0 <- c(ry0,(exp(coefs[2]*0+coefs[1])/(1+exp(coefs[2]*0+coefs[1]))))

}

#output binomial GLM fitting results for pfr1

write.table(data.frame(ra,rb,rx5,rx95,ry0), "hssn-random1.txt")

#similar procedures for hssn-random3.txt, hssn-random5.txt, hssn-random7.txt and hssn-random9.txt"

Appendix S4 – **ImageJ macro code to batch extract distribution area from maps (ImageJ Macro Code (DistributionArea).txt)**

//Batch Macro Code

dir = getDirectory("Choose a Directory");

setBatchMode(true);

img = getFileList(dir);

for(i=0; i<lengthOf(img); i=i+1)

{

open(img[i]);

setAutoThreshold("Default");

//run("Threshold...");

setThreshold(30, 50);

setOption("BlackBackground", false);

run("Convert to Mask");

run("Analyze Particles...", "size=0-Infinity circularity=0.00-1.00 show=Nothing clear summarize");

saveAs("Text", "E:\\apweb\\summ\\Summary.xls");

}

setBatchMode(false);

Appendix S5 – **Prediction of plant utilization probability at global scale when species number in a plant family as plant apparency**

| Utilization group | Consumer type# | *a* | *G* | *P*(*a* = 0) | *b* |
| --- | --- | --- | --- | --- | --- |
| Agrilus | bark borers | 0.0004 | 32.5 | **0.0000** | -1.85 |
| longicorns | wood borers | 0.0031 | 135.7 | **0.0000** | -0.95 |
| Tephritidae | fruit eaters | 0.0001 | 10.9 | **0.0010** | -1.96 |
| Cecidomyiidae | gallers | 0.0032 | 174.6 | **0.0000** | -1.63 |
| food | humans | 0.0024 | 148.2 | **0.0000** | -1.65 |
| medicines | humans | 0.0078 | 215.6 | **0.0000** | -1.36 |
| environmental uses | humans | 0.0046 | 152.3 | **0.0000** | -0.85 |
| food additives | humans | 0.0013 | 103.1 | **0.0000** | -1.86 |
| forages | humans | 0.0003 | 24.6 | **0.0000** | -2.33 |
| invertebrate food | humans | 0.0001 | 5.4 | **0.0207** | -3.45 |
| gene sources | humans | 0.0015 | 122.2 | **0.0000** | -2.29 |
| fuels | humans | 0.0003 | 24.9 | **0.0000** | -2.21 |
| vertebrate poisons | humans | 0.0009 | 66.2 | **0.0000** | -1.46 |
| weeds | humans | 0.0009 | 65.5 | **0.0000** | -1.18 |
| social uses | humans | 0.0005 | 38.2 | **0.0000** | -2.57 |
| CITES endangered plants | humans | 0.0005 | 36.5 | **0.0000** | -2.52 |
| non- vertebrate poisons | humans | 0.0002 | 19.7 | **0.0000** | -2.88 |
| materials | humans | 0.0018 | 104.4 | **0.0000** | -1.02 |
| harmful organism hosts | humans | 0.0004 | 34.4 | **0.0000** | -3.05 |
| bee plants | humans | 0.0002 | 18.0 | **0.0000** | -2.60 |
| extrafloral nectaries | bodyguard predators | 0.0011 | 86.2 | **0.0000** | -1.64 |
| Cassidinae | leaf eaters | 0.0004 | 32.2 | **0.0000** | -2.85 |
| Tischeriidae | leaf miners | 0.0002 | 14.1 | **0.0002** | -3.30 |
| leaf-mining Chrysomelidae | leaf miners | 0.0018 | 138.4 | **0.0000** | -2.15 |
| Gracillariidae | leaf miners | 0.0004 | 31.3 | **0.0000** | -1.40 |
| Agromyzidae | leaf miners | 0.0023 | 142.6 | **0.0000** | -1.54 |
| Tortricidae | leaf rollers | 0.0042 | 163.2 | **0.0000** | -1.09 |
| Chalcidoidea | parasitoids | 0.0047 | 190.5 | **0.0000** | -1.41 |
| nematodes | pathogens | 0.0067 | 208.1 | **0.0000** | -1.37 |
| virus | pathogens | 0.0024 | 167.8 | **0.0000** | -2.17 |
| fungi | pathogens | 0.0038 | 101.3 | **0.0000** | -0.19 |
| dioecy | pollinators | 0.0008 | 51.9 | **0.0000** | -0.77 |
| gynomonoecy | pollinators | 0.0002 | 15.4 | **0.0001** | -2.60 |
| andromonoecy | pollinators | 0.0001 | 9.0 | **0.0026** | -2.64 |
| monoecy | pollinators | 0.0001 | 9.8 | **0.0018** | -1.51 |
| hermaphrodite | pollinators | 0.0009 | 54.0 | **0.0000** | -0.82 |
| gynodioecy | pollinators | 0.0008 | 60.7 | **0.0000** | -2.03 |
| polygamodioecy | pollinators | 0.0006 | 44.7 | **0.0000** | -2.06 |
| polygamomonoecy | pollinators | 0.0004 | 30.2 | **0.0000** | -1.84 |
| androdioecy | pollinators | 0.0003 | 23.3 | **0.0000** | -2.84 |
| aphids | sap suckers | 0.0080 | 166.8 | **0.0000** | -0.74 |
| whiteflies | sap suckers | 0.0005 | 41.4 | **0.0000** | -1.67 |
| plant hoppers | sap suckers | 0.0008 | 58.7 | **0.0000** | -1.25 |
| psyllids | sap suckers | 0.0023 | 127.2 | **0.0000** | -1.17 |
| spider mites | sap suckers | 0.0054 | 196.5 | **0.0000** | -1.36 |
| arbuscular mycorrhizal fungi | mutualists | 0.0007 | 54.1 | **0.0000** | -1.88 |

A binomial GLM with logit link (*UP* = exp(*a* × *PA* + *b*)/(exp(*a* × *PA* + *b*)+ 1)) to predict utilization probability (*UP*) as a function of plant apparency (*PA*; species number in a plant family here). See text for details. # —utilizer type were based on most members of the corresponding utilization group. *P*(*a* = 0) in bold indicates that the slope *a* is significantly different from 0 (*P* < 0.05).

Appendix S6 – **Prediction of plant utilization probability at global scale when distribution area of a plant family as plant apparency**

| Utilization group | Consumer type# | *a* | *G* | *P*(*a* = 0) | *b* |
| --- | --- | --- | --- | --- | --- |
| Agrilus | bark borers | 1.15E-07 | 76.3 | **0.0000** | -2.70 |
| longicorns | wood borers | 1.32E-07 | 99.3 | **0.0000** | -1.07 |
| Tephritidae | fruit eaters | 8.72E-08 | 43.2 | **0.0000** | -2.55 |
| Cecidomyiidae | gallers | 1.83E-07 | 163.6 | **0.0000** | -2.00 |
| food | humans | 1.54E-07 | 134.8 | **0.0000** | -2.00 |
| medicines | humans | 2.79E-07 | 214.1 | **0.0000** | -1.73 |
| environmental uses | humans | 2.14E-07 | 157.9 | **0.0000** | -1.20 |
| food additives | humans | 1.33E-07 | 107.8 | **0.0000** | -2.37 |
| forages | humans | 1.26E-07 | 70.0 | **0.0000** | -3.52 |
| invertebrate food | humans | 8.25E-08 | 12.1 | **0.0005** | -4.24 |
| gene sources | humans | 1.34E-07 | 103.6 | **0.0000** | -2.70 |
| fuels | humans | 1.05E-07 | 53.6 | **0.0000** | -3.07 |
| vertebrate poisons | humans | 1.88E-07 | 174.0 | **0.0000** | -2.65 |
| weeds | humans | 2.45E-07 | 225.4 | **0.0000** | -2.65 |
| social uses | humans | 1.11E-07 | 53.7 | **0.0000** | -3.39 |
| CITES endangered plants | humans | 8.84E-08 | 35.2 | **0.0000** | -3.03 |
| non- vertebrate poisons | humans | 1.08E-07 | 36.5 | **0.0000** | -3.90 |
| materials | humans | 1.23E-07 | 94.7 | **0.0000** | -1.29 |
| harmful organism hosts | humans | 1.89E-07 | 90.2 | **0.0000** | -5.42 |
| bee plants | humans | 1.11E-07 | 45.3 | **0.0000** | -3.67 |
| extrafloral nectaries | bodyguard predators | 1.18E-07 | 91.3 | **0.0000** | -2.07 |
| Cassidinae | leaf eaters | 1.34E-07 | 62.3 | **0.0000** | -4.11 |
| Tischeriidae | leaf miners | 1.02E-07 | 23.2 | **0.0000** | -4.31 |
| leaf-mining Chrysomelidae | leaf miners | 1.57E-07 | 136.2 | **0.0000** | -2.69 |
| Gracillariidae | leaf miners | 1.03E-07 | 71.6 | **0.0000** | -2.05 |
| Agromyzidae | leaf miners | 1.91E-07 | 172.0 | **0.0000** | -2.08 |
| Tortricidae | leaf rollers | 1.84E-07 | 145.5 | **0.0000** | -1.32 |
| Chalcidoidea | parasitoids | 1.89E-07 | 159.6 | **0.0000** | -1.62 |
| nematodes | pathogens | 2.46E-07 | 196.9 | **0.0000** | -1.68 |
| virus | pathogens | 1.86E-07 | 170.6 | **0.0000** | -2.77 |
| fungi | pathogens | 2.38E-07 | 134.4 | **0.0000** | -0.62 |
| dioecy | pollinators | 8.30E-08 | 51.2 | **0.0000** | -1.01 |
| gynomonoecy | pollinators | 1.20E-07 | 52.8 | **0.0000** | -3.83 |
| andromonoecy | pollinators | 1.20E-07 | 57.2 | **0.0000** | -3.69 |
| monoecy | pollinators | 9.70E-08 | 61.6 | **0.0000** | -2.20 |
| hermaphrodite | pollinators | 1.33E-07 | 104.2 | **0.0000** | -1.26 |
| gynodioecy | pollinators | 1.66E-07 | 137.3 | **0.0000** | -3.24 |
| polygamodioecy | pollinators | 1.24E-07 | 84.4 | **0.0000** | -2.92 |
| polygamomonoecy | pollinators | 1.16E-07 | 80.8 | **0.0000** | -2.59 |
| androdioecy | pollinators | 1.27E-07 | 55.4 | **0.0000** | -4.02 |
| aphids | sap suckers | 3.36E-07 | 201.5 | **0.0000** | -1.24 |
| whiteflies | sap suckers | 1.08E-07 | 75.2 | **0.0000** | -2.30 |
| plant hoppers | sap suckers | 1.39E-07 | 117.7 | **0.0000** | -1.99 |
| psyllids | sap suckers | 1.70E-07 | 144.9 | **0.0000** | -1.64 |
| spider mites | sap suckers | 1.94E-07 | 160.0 | **0.0000** | -1.53 |
| arbuscular mycorrhizal fungi | mutualists | 1.71E-07 | 149.1 | **0.0000** | -3.01 |

A binomial GLM with logit link (*UP* = exp(*a* × *PA* + *b*)/(exp(*a* × *PA* + *b*)+ 1)) to predict utilization probability (*UP*) as a function of plant apparency (*PA*; species number in a plant family here). See text for details. # —utilizer type were based on most members of the corresponding utilization group. *P*(*a* = 0) in bold indicates that the slope *a* is significantly different from 0 (*P* < 0.05).

Appendix S7 – **Prediction of plant utilization probability at global scale when phylogenetic closeness to common plant families as plant phylogenetic closeness**

| Utilization group | Consumer type# | *a* | *G* | *P*(*a* = 0) | *b* |
| --- | --- | --- | --- | --- | --- |
| Agrilus | bark borers | -0.0089 | 26.3 | **0.0000** | -0.48 |
| longicorns | wood borers | -0.0070 | 27.4 | **0.0000** | 0.77 |
| Tephritidae | fruit eaters | -0.0102 | 27.8 | **0.0000** | -0.68 |
| Cecidomyiidae | gallers | -0.0099 | 47.5 | **0.0000** | 0.64 |
| food | humans | -0.0103 | 49.9 | **0.0000** | 0.55 |
| medicines | humans | -0.0065 | 23.8 | **0.0000** | 0.74 |
| environmental uses | humans | -0.0052 | 15.7 | **0.0001** | 0.74 |
| food additives | humans | -0.0104 | 43.7 | **0.0000** | 0.12 |
| forages | humans | -0.0131 | 37.1 | **0.0000** | -0.66 |
| invertebrate food | humans | -0.0139 | 14.2 | **0.0002** | -1.95 |
| gene sources | humans | -0.0100 | 36.4 | **0.0000** | -0.18 |
| fuels | humans | -0.0103 | 26.6 | **0.0000** | -0.78 |
| vertebrate poisons | humans | -0.0096 | 40.6 | **0.0000** | 0.21 |
| weeds | humans | -0.0085 | 34.9 | **0.0000** | 0.35 |
| social uses | humans | -0.0085 | 16.2 | **0.0001** | -1.14 |
| CITES endangered plants | humans | -0.0106 | 24.8 | **0.0000** | -0.92 |
| non- vertebrate poisons | humans | -0.0186 | 41.6 | **0.0000** | -0.91 |
| materials | humans | -0.0078 | 32.3 | **0.0000** | 0.63 |
| harmful organism hosts | humans | -0.0225 | 55.7 | **0.0000** | -0.68 |
| bee plants | humans | -0.0099 | 17.7 | **0.0000** | -1.27 |
| extrafloral nectaries | bodyguard predators | -0.0080 | 28.0 | **0.0000** | -0.04 |
| Cassidinae | leaf eaters | -0.0135 | 29.1 | **0.0000** | -1.05 |
| Tischeriidae | leaf miners | -0.0108 | 11.4 | **0.0007** | -1.91 |
| leaf-mining Chrysomelidae | leaf miners | -0.0106 | 44.0 | **0.0000** | 0.08 |
| Gracillariidae | leaf miners | -0.0063 | 17.0 | **0.0000** | -0.33 |
| Agromyzidae | leaf miners | -0.0091 | 40.6 | **0.0000** | 0.47 |
| Tortricidae | leaf rollers | -0.0069 | 26.7 | **0.0000** | 0.75 |
| Chalcidoidea | parasitoids | -0.0068 | 25.6 | **0.0000** | 0.57 |
| nematodes | pathogens | -0.0101 | 52.6 | **0.0000** | 1.15 |
| virus | pathogens | -0.0130 | 66.6 | **0.0000** | 0.48 |
| fungi | pathogens | -0.0028 | 4.3 | **0.0371** | 0.86 |
| dioecy | pollinators | -0.0042 | 9.9 | **0.0017** | 0.16 |
| gynomonoecy | pollinators | -0.0092 | 14.9 | **0.0001** | -1.37 |
| andromonoecy | pollinators | -0.0083 | 11.3 | **0.0008** | -1.56 |
| monoecy | pollinators | -0.0043 | 7.1 | **0.0079** | -0.86 |
| hermaphrodite | pollinators | -0.0037 | 7.9 | **0.0050** | 0.07 |
| gynodioecy | pollinators | -0.0128 | 51.5 | **0.0000** | -0.07 |
| polygamodioecy | pollinators | -0.0102 | 32.0 | **0.0000** | -0.46 |
| polygamomonoecy | pollinators | -0.0071 | 16.9 | **0.0000** | -0.69 |
| androdioecy | pollinators | -0.0115 | 20.2 | **0.0000** | -1.31 |
| aphids | sap suckers | -0.0052 | 14.9 | **0.0001** | 0.98 |
| whiteflies | sap suckers | -0.0083 | 26.4 | **0.0000** | -0.30 |
| plant hoppers | sap suckers | -0.0089 | 37.2 | **0.0000** | 0.29 |
| psyllids | sap suckers | -0.0074 | 29.1 | **0.0000** | 0.54 |
| spider mites | sap suckers | -0.0098 | 49.2 | **0.0000** | 1.03 |
| arbuscular mycorrhizal fungi | mutualists | -0.0105 | 38.7 | **0.0000** | -0.18 |

A binomial GLM with logit link (*UP* = exp(*a* × *PA* + *b*)/(exp(*a* × *PA* + *b*)+ 1)) to predict utilization probability (*UP*) as a function of phylogenetic closeness (*PD_min_*). See text for details. # —utilizer type were based on most members of the corresponding utilization group. *P*(*a* = 0) in bold indicates that the slope *a* is significantly different from 0 (*P* < 0.05).

Appendix S8 – **Phylogenetic signal (*D*) of utilization presence-absence in world plant families**

|  | Utilization group | Consumer type | *HF* | *D* | *P_random_* | *P_Brownian_* |
| --- | --- | --- | --- | --- | --- | --- |
| 1 | Agrilus | bark borers | 75 | 0.951 | 0.333 | **0** |
| 2 | longicorns | wood borers | 193 | 0.784 | **0.004** | **0** |
| 3 | Tephritidae | fruit eaters | 58 | 0.773 | **0.027** | **0** |
| 4 | Cecidomyiidae | gallers | 149 | 0.802 | **0.005** | **0** |
| 5 | food | humans | 136 | 0.885 | **0.079** | **0** |
| 6 | medicines | humans | 197 | 0.841 | **0.021** | **0** |
| 7 | environmental uses | humans | 214 | 0.808 | **0.006** | **0** |
| 8 | food additives | humans | 103 | 0.881 | **0.089** | **0** |
| 9 | forages | humans | 48 | 1.043 | 0.633 | **0** |
| 10 | invertebrate food | humans | 15 | 0.987 | 0.500 | **0.011** |
| 11 | gene sources | humans | 86 | 0.929 | 0.215 | **0** |
| 12 | fuels | humans | 53 | 0.998 | 0.504 | **0** |
| 13 | vertebrate poisons | humans | 116 | 0.888 | 0.108 | **0** |
| 14 | weeds | humans | 138 | 0.876 | **0.061** | **0** |
| 15 | social uses | humans | 45 | 0.949 | 0.356 | **0** |
| 16 | CITES endangered plants | humans | 46 | 0.967 | 0.398 | **0** |
| 17 | non- vertebrate poisons | humans | 29 | 0.935 | 0.359 | **0** |
| 18 | materials | humans | 171 | 0.749 | **0.002** | **0** |
| 19 | harmful organism hosts | humans | 30 | 1.392 | 0.987 | **0** |
| 20 | bee plants | humans | 36 | 1.067 | 0.657 | **0** |
| 21 | extrafloral nectaries | bodyguard predators | 111 | 0.756 | **0.001** | **0** |
| 22 | Cassidinae | leaf eaters | 34 | 0.943 | 0.362 | **0** |
| 23 | Tischeriidae | leaf miners | 19 | 1.219 | 0.816 | **0** |
| 24 | Leaf-mining Chrysomelidae | leaf miners | 99 | 0.957 | 0.320 | **0** |
| 25 | Gracillariidae | leaf miners | 104 | 0.898 | 0.123 | **0** |
| 26 | Agromyzidae | leaf miners | 142 | 0.915 | 0.150 | **0** |
| 27 | Tortricidae | leaf rollers | 193 | 0.966 | 0.309 | **0** |
| 28 | Chalcidoidea | parasitoids | 176 | 0.851 | **0.034** | **0** |
| 29 | nematodes | pathogens | 191 | 0.686 | **0.000** | **0** |
| 30 | virus | pathogens | 109 | 0.840 | **0.032** | **0** |
| 31 | fungi | pathogens | 260 | 0.854 | **0.032** | **0** |
| 32 | dioecy | pollinators | 170 | 0.926 | 0.171 | **0** |
| 33 | gynomonoecy | pollinators | 35 | 1.220 | 0.908 | **0** |
| 34 | andromonoecy | pollinators | 32 | 1.308 | 0.963 | **0** |
| 35 | monoecy | pollinators | 83 | 0.970 | 0.386 | **0** |
| 36 | hermaphrodite | pollinators | 166 | 0.868 | **0.044** | **0** |
| 37 | gynodioecy | pollinators | 77 | 1.012 | 0.560 | **0** |
| 38 | polygamodioecy | pollinators | 69 | 1.047 | 0.674 | **0** |
| 39 | polygamomonoecy | pollinators | 74 | 1.061 | 0.718 | **0** |
| 40 | androdioecy | pollinators | 31 | 1.223 | 0.907 | **0** |
| 41 | aphids | sap suckers | 239 | 0.822 | **0.007** | **0** |
| 42 | whiteflies | sap suckers | 90 | 0.866 | **0.089** | **0** |
| 43 | plant hoppers | sap suckers | 129 | 0.712 | **0.003** | **0** |
| 44 | psyllids | sap suckers | 167 | 0.722 | **0.000** | **0** |
| 45 | spider mites | sap suckers | 184 | 0.792 | **0.002** | **0** |
| 46 | arbuscular mycorrhizal fungi | mutualists | 83 | 1.025 | 0.597 | 0 |

*P_random_* and *P_Brownian_* are *P*-values indicating whether the estimate of phylogenetic signal (*D*) is significantly different from random (*D* = 1) or from Brownian expectation (*D* = 0), respectively. Number of randomizations = 1000. *HF* is the number of host plant families. A number in bold indicated a significant difference for *P*-values (*P* < 0.1). See text for details.
